# Male External Catheters and Urinary Tract Infections: A Systematic Review and Meta-Analysis of Current Evidence and the Need for Standardized Reporting

**DOI:** 10.7759/cureus.90264

**Published:** 2025-08-17

**Authors:** Francisco S Mercado, Michaela M Kop, Ana Danko, Heather Zimmerman, Colby Watase

**Affiliations:** 1 Medicine, Tripler Army Medical Center, Honolulu, USA; 2 Medicine, University of Hawaii John A. Burns School of Medicine, Honolulu, USA

**Keywords:** ca-uti, condom catheter, male external catheter, systematic review and meta analysis, urinary tract infection (uti)

## Abstract

Catheter-associated urinary tract infections (CAUTIs) are a significant cause of morbidity and mortality among hospitalized patients, prompting the implementation of various strategies to reduce their incidence, including the use of male external catheters (MEC). However, the effectiveness of MEC in preventing urinary tract infections (UTIs) remains uncertain. This study aimed to systematically summarize existing research and conduct a meta-analysis to evaluate the impact of MEC on UTI rates and related outcomes. We searched PubMed, Medline, Embase, ClinicalTrials.gov, and the WHO International Clinical Trials Registry Platform for peer-reviewed articles and clinical trials from inception until June 30, 2024, ultimately including 42 of 1,545 publications in our systematic review, comprising 40 observational studies and two randomized controlled trials (RCTs). Our meta-analysis focused on eight articles with complete methodology and outcomes, revealing a non-significant 27% decrease in UTIs among MEC users (odds ratio 0.73, confidence interval 0.44-1.22) with moderate heterogeneity (I^2^ = 58.47%). Additionally, asymptomatic bacteriuria was associated with a non-significant 3% lower risk for MEC (odds ratio 0.97, confidence interval 0.61-1.54) and mild heterogeneity (I^2^ = 29.79%). The incidence rate ratio (IRR) for UTIs significantly decreased when comparing MEC users to indwelling urinary catheter (IUC) users (odds ratio, 0.20; 95% confidence interval, -0.03 to 0.43), demonstrating significant heterogeneity (I^2^ = 99.98%). Our findings indicated variability in the reporting of secondary outcomes, with a non-significant decrease in UTI and asymptomatic bacteriuria events among MEC users compared to IUC users. However, effect sizes were associated with significant heterogeneity. We recommend standardized reporting of secondary outcomes in future studies to enhance comparability and reliability.

## Introduction and background

Catheter-associated urinary tract infection (CAUTI) is the leading cause of nosocomial infections and a major contributor to sepsis [1,2]. These debilitating infections are responsible for approximately one million cases of hospital-acquired infections in the United States each year [2]. Additionally, these preventable infections impose a growing financial strain on the US healthcare system, with associated costs ranging from $115 million to $1.82 billion annually [2]. Given the high prevalence, substantial economic burden, and considerable health impacts associated with CAUTI, it is imperative to implement evidence-based preventative strategies to mitigate its effects and improve patient outcomes.

The Infectious Disease Society of America recommends the judicious use of indwelling urinary catheters (IUC) to mitigate the burden of CAUTI [3]. Similarly, the 2009 Centers for Disease Control and Prevention Guidelines advocate the use of external catheters as an alternative to IUC [4]. In response, external urinary catheters for both males and females have emerged as a preventative measure to combat CAUTI. Male external catheters (MECs) have been used since the 1600s [5]. Since then, contemporary designs and methodologies (latex and non-latex sheathed devices, retracted penile pouches, and glans-adherent devices) have been developed to reduce discomfort and enhance usability [6,7]. Likewise, female external catheters (FECs) have undergone rapid development and have gained widespread use in both acute and outpatient settings [8]. More recently, in 2024, an innovative systematic review and meta-analysis focusing on FEC systems showed significant reductions in IUC utilization rates for nurse-driven protocols and non-significant reductions in CAUTI rates [9].

While recent systematic evidence underscores the advantages of FEC in preventing UTI, the effectiveness of their male counterparts remains uncertain. This systematic review and meta-analysis investigate the relationship between MEC, UTI, and other secondary outcomes (i.e., asymptomatic bacteriuria (AB), incidence rate ratio (IRR), bacterial isolates, dermatologic manifestations, etc.), aiming to provide a comprehensive overview of MEC's role in UTI prevention, thereby informing clinical practice and guiding future research.

## Review

Methodology

Search Strategy

A comprehensive search of PubMed, Medline, Embase, ClinicalTrials.gov, and the WHO International Clinical Trials Registry Platform (ICTRP) was conducted from their inception to June 30, 2024, following the Preferred Reporting Items for Systematic Reviews and Meta-Analyses (PRISMA) guidelines. The study protocol was registered in PROSPERO (ID: CRD42024557828). Our search strategy employed keywords such as "condom catheter," "male wicking device," "male pure wick," "penile sheath," "male external urine device," "male external wicking," "CAUTI," "Catheter-associated urinary tract infection," "UTI," "urinary tract infection," "bacteriuria," "pyuria," "acute pyelonephritis," "acute prostatitis," and "acute cystitis." The resulting list of articles from each database was uploaded to the Covidence platform (Covidence systematic review software, Veritas Health Innovation, Melbourne, Australia), which was used for article screening [10].

Study Selection

The inclusion criteria were randomized controlled trials (RCTs), observational studies (retrospective and prospective cohort, case-control, prevalence studies), case reports involving study populations of adults (aged 18 years or older), studies with UTI and CAUTI explicitly defined in the methodology, peer-reviewed abstracts, and studies written in the English language. In contrast, the exclusion criteria were rapid reviews, narrative reviews, books, opinions, editorials, duplicate articles, unavailable texts, retractions, consensus documents, and studies with unclear methods and outcomes.

Data Extraction and Quality Assessment

Given the lack of a formally established definition for UTI related to MEC, we defined UTI as the primary outcome. For this review, a UTI occurring in patients using indwelling urinary catheters is referred to as a CAUTI, while a UTI in patients using MECs is referred to as MEC-UTI [11]. Secondary outcomes included AB, CAUTI or MEC-UTI per 1000 patient-days, CAUTI or MEC-UTI per 1000 device-days, IRR, IUC utilization rate, pain, skin breakdown or manifestations, pressure injury, length of stay (LOS), cost-effectiveness, device utilization ratio (DUR), standard infection ratio, and standard utilization ratio (SUR).

FM and MK utilized the Covidence Platform to screen for articles. Any disagreements regarding the inclusion of an article were independently resolved by either CW, AD, or HZ. FM and MK independently extracted data from the final set of 42 articles. Ultimately, they compared their data and reached a consensus to finalize the data collectively. FM and MK conducted a quality assessment for the eight articles included in the meta-analysis. The Covidence platform was used to evaluate the quality of the two RCTs, whereas the Risk of Bias in Non-randomized Studies of Interventions (ROBINS-I) tool was used for the six observational studies [12]. Two authors, FM and MK, independently conducted data extraction using the ROBINS-I for "risk of bias" assessment, and disagreements were resolved by discussion [12].

Data Synthesis and Analysis

We conducted a meta-analysis by gathering data from two RCTs and six observational studies, focusing on UTI and AB events, and the IRR between MEC and IUC (IRR) as outcomes, as these articles provide detailed methodologies and results. A random-effects model was used to determine the pooled effects of UTI and AB. We used I^2^ to assess heterogeneity [13]. UTI and AB events are presented as odds ratios (OR) in the forest plots. Secondary outcomes not included in the meta-analysis were systematically summarized in the tables. Given that only eight studies were included in the meta-analysis, it was not possible to determine the presence of publication bias. Stata/BE version 18 (StataCorp LLC, College Station, TX, USA) was used for meta-analysis.

Results

**Figure 1:**
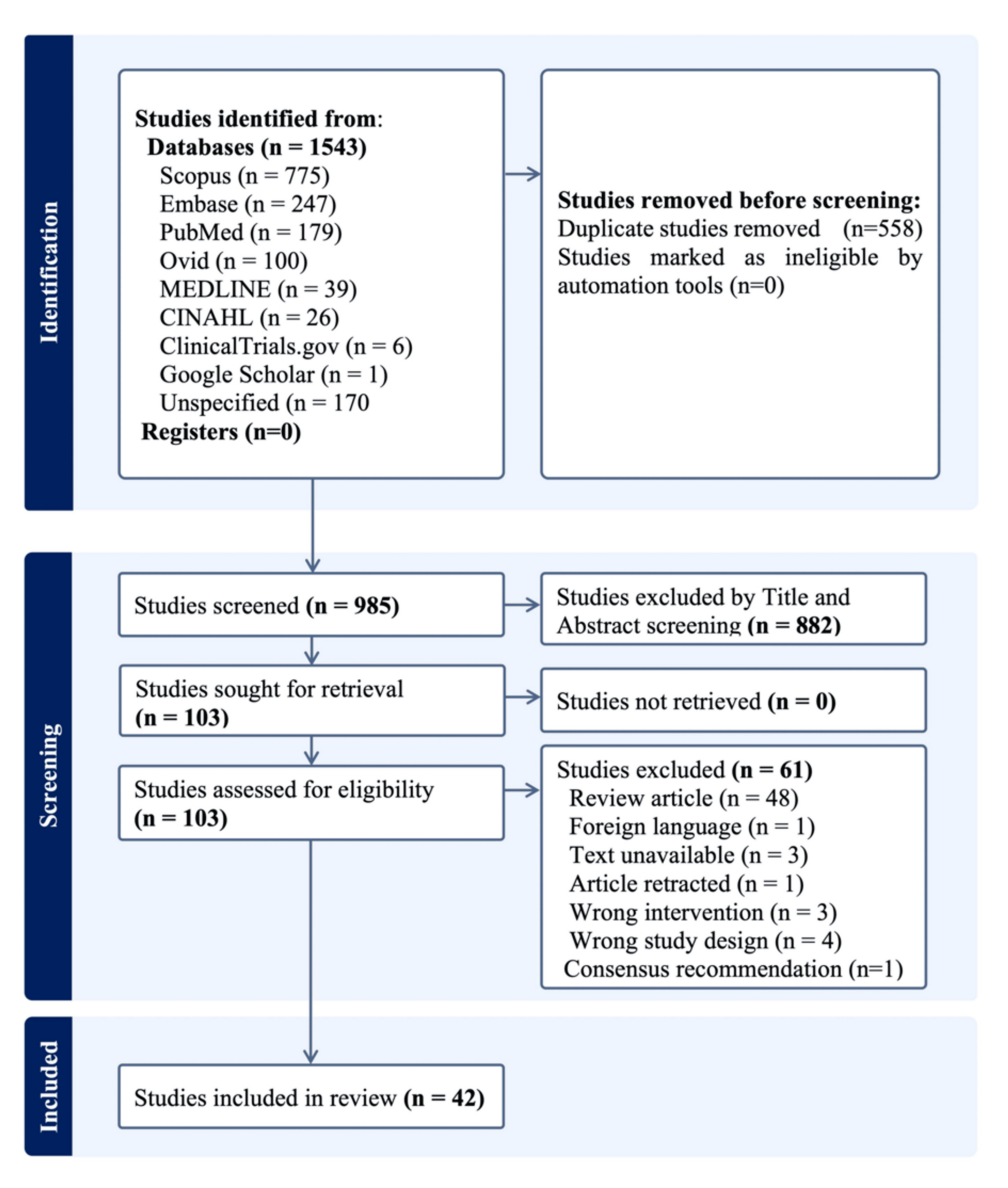
PRISMA flow diagram indicating the study selection and inclusion process. PRISMA: Preferred Reporting Items for Systematic Reviews and Meta-Analyses

Our search strategy identified 42 articles for inclusion in this review (Figure 1, PRISMA diagram). These articles comprised two RCTs, 13 prospective cohort studies, eight retrospective cohort studies, nine nurse-driven pre-post studies, five prevalence studies, and five case reports. Of these, eight studies with complete manuscripts and outcomes related to UTI and AB events, as well as the IRR, were incorporated into the meta-analysis and risk of bias (ROB) assessment.

Study Characteristics

Table 1 summarizes the included studies, which were predominantly conducted in the United States (76%, 32/42) and exhibited extensive variability in settings and patient characteristics. Numerous studies have included spinal cord patients [14-23]. Diverse exclusion criteria were noted, such as progressive spinal cord injury (SCI), congenital spinal conditions, urinary retention, or bladder obstruction [22]. Some studies included individuals older than 18 years, while one study included men older than 40 years [15,22,24]. Patient age in the meta-analyzed studies ranged widely (mean 45.9 to 73.7 years), indicating broad target populations [22,24]. Studies spanned diverse healthcare settings, from community hospitals to spinal cord services, reflecting widespread application but also complicating generalization.

**Table 1:** Studies included in the systematic review. CAABU: catheter-associated asymptomatic bacteriuria; CAUTI: catheter-associated urinary tract infection; CI: confidence interval; GFR: glomerular filtration rate; ISC: intermittent self-catheterization; HAPI: hospital-acquired pressure injury; IUC: indwelling urinary catheter; MEC: male external catheter; OR: odds risk; MDR: multiple-drug resistant; SCI: spinal cord injury; SPC: suprapubic catheterization; UTI: urinary tract infection; SUR: standard utilization rate; t: t statistics

Study identifier	Country	Setting	Design	Total sample size (N)	Outcome
Cameron 2009 [14]	USA	Registry database	Prevalence study	24,762	The MEC usage fell from 1972 to 2001. IUC usage increased in 2001. Only 34.6% of individuals maintained MEC at discharge.
Ruz 2000 [15]	Spain	Acute care	Prospective cohort study	128	The UTI in IUC is 2.72 events/100 person-days, while in MEC is 0.24 events/100 person-days.
Gao 2017 [16]	USA	Outpatient clinic	Retrospective cohort study	43	Male gender, cervical injury, and MEC use were associated with complications, including UTI and renal insufficiency.
Gilmore 1992 [17]	USA	Acute care	Prospective cohort study	119	There was a high incidence of urethral, perineal, rectal, and drainage bag colonization with *Pseudomonas* and *Klebsiella* in SCI patients. Removing the MECs at night reduced urethral colonization but not perineal or rectal colonization with *Pseudomonas*.
Hennessey 2019 [18]	Australia	Acute care	Retrospective cohort study	143	Substitution of IUC with either ISC or SPC was associated with a reduced UTI rate. There was no UTI noted for the MEC patient.
Montgomerie 1980 [19]	USA	Acute care	Prospective cohort study	20	MEC is associated with an increased risk of *Pseudomonas* colonization in the urethra (t = 3.25; p< 0.025). Reservoirs include the MEC, the urethra, the perineum, and the rectum.
Myers 2018 [20]	USA	Registry/database	Prospective cohort study	611	MEC users have better voiding and higher satisfaction than intermittent IUC users.
Roth 2019 [21]	USA	Outpatient clinic	Retrospective cohort study	1282	UTI frequency is higher than with pads/condoms, and the odds of hospitalization are highest with IUC.
Singh 2011 [22]	India	Acute care	Prospective cohort study	545	MEC patients had fewer urethral strictures than IUC patients (P = 0.04), but they had more incontinence (P < 0.001) than the intermittent catheterization and normal voiding group. MEC is associated with increased residual volume and incontinence.
Waites 2000 [23]	USA	Long-term care facility	Retrospective cohort study	287	Polymicrobial bacteriuria and MDR *Pseudomonas* colonization are more common in MEC patients.
Saint 2006 [24]	USA	Acute care, long-term care facility, outpatient clinic	Randomized controlled trial	76	The incidence of adverse outcomes was 131/1000 patient-days in IUC, while it was 70/1000 patient-days in MEC. Patients without dementia who had IUC were five times likely to have bacteriuria, symptomatic UTI, or to die. Patients with MEC were more comfortable.
Chirca 2018 [25]	USA	Acute care	Nurse-driven pre-post study	N/A	From 2013 to 2017, the average urinary catheter utilization rate decreased from 23.7% to 14.5%, while the average incidence of CAUTI per 1,000 catheter days dropped from 1.99 to 0.8, and the total number of CAUTIs reported each year decreased from 52 in both 2013 and 2014 to 15 in 2017.
Fields 2019 [26]	USA	Acute care	Nurse-driven pre-post study	N/A	The implementation of the Intermittent Catheterization Algorithm resulted in a significant reduction in the CAUTI rate, from 4.5±3.4 to 0.42±1.45 (p=0.0081), and a decrease in the Device Utilization ratio, from 0.8±0.08 to 0.53±0.09 (p<0.0001).
Ghonim 2020 [27]	USA	Acute care	Nurse-driven pre-post study	N/A	CAUTIs decreased from 51 in 2017 to 29 in 2018, and then to 15 in 2019.
Mayes 2020 [28]	USA	Acute care	Nurse-driven pre-post study	N/A	IUC utilization rate decreased from 56% in 2017 to 49% in 2018. CAUTI per 1000 device-days decreased from 1.29 in 2017 to 0 in 2018. Hospital-acquired pressure injuries per 1000 device-days decreased from 4 in 2017 to 0 in 2018.
Saint 2019 [29]	USA	Acute care	Prospective cohort study	80	MEC patients are significantly less likely than IUC patients to report complications during catheter placement (13.9% vs. 43.2%, P < 0.001).
Zaghbib 2019 [30]	Tunisia	Acute care	Case report	N/A	A 58-year-old male with schizophrenia using a condom catheter for urinary incontinence secured with a rubber band, which resulted in penile necrosis.
Sinha 2018 [31]	India	Acute care	Case report	N/A	Penile skin erosion was seen in an MEC user.
Morrissey 1985 [32]	USA	Acute care	Case report	N/A	A 58 y/o neutropenic man developed multiple penile ulcers due to candida infection after MEC use.
Alshahri 2024 [33]	Saudi Arabia	Outpatient clinic	Cross-sectional study	385	*Escherichia coli *and *Proteus mirabilis* are the most common in IUC, while *E. coli *and *Pseudomonas* are the most common in MEC.
Fierer 1981 [34]	USA	Acute care	Prospective cohort study	52	MEC patient urinals/urinary drainage bags were colonized by *Pseudomonas stuartii.*
Johnson 1983 [35]	USA	Long-term care facility	Retrospective cohort study	84	MEC is associated with a higher rate of infection. Common pathogens included *Proteus*, *Pseudomonas*, *E. coli*, and *Klebsiella*. MEC was associated with penile strangulation and skin ulceration.
Moore 2024 [36]	USA	Acute care	Nurse-driven pre-post study	N/A	There was an increase in utilization of the device from 512 (January-March 2023) to 1352 (July-September 2023). While the standard infection ratio was 0.59 (infection rate: 1050/1000 line days), it improved to 0.41 (infection rate: 0.73/1000 line days).
Grigoryan 2014 [37]	USA	Acute care	Prevalence study	308	Gram-positive infections are more common in patients using MEC, while *Pseudomonas* and *Candida* infections are more common in patients using IUC. The mean number of organisms/patients was higher in patients with MEC. *Enterococci* and *Enterobacteriaceae* were equally prevalent in both types of catheters.
Barash 2021 [38]	USA	Acute care	Case report	N/A	The patient used a MEC during extended cave diving (15 hours), which contributed to colonization by *Aeromonas*, causing acute prostatitis.
Hirsh 1979 [39]	USA	Acute care	Prospective cohort study	94	Uncooperative patients have 8 (53.3%; N = 15) UTIs in 9.6 days after MEC placement, compared to cooperative patients, who have 0 (0%; N = 79). Simple catheterization after MEC may be associated with UTI.
Zimakoff 1996 [40]	Denmark	Acute care, long-term care facility	Prevalence study	3665	MEC OR 5.94 (2.83-12.48; p < 0.001) is associated with higher rates of infection compared to IUC OR 3.34 (2.32-4.80; p)
Hebel 1990 [41]	USA	Long-term care facility	Prevalence study	4,259	In men, MEC is associated with decubitus ulcer (V 0.073) compared to IUC (V 0.020). Functional dependence is associated with both MEC and IUC.
Blake 2024 [42]	USA	Acute care	Nurse-driven pre-post study	N/A	IUC SUR improved from 0.81 to 0.58 (p < 0.001), with 1 CAUTI occurring in 7 months compared to 4 in 3 months.
Cope 2009 [43]	USA	Acute care	Retrospective cohort study	N/A	53 (32%) of CAABU patients were inappropriately treated with antibiotics.
Gao 2016 [44]	USA	N/A	Retrospective cohort study	24	54% of MEC users had bladder stones. 80% of IUC users had hydronephrosis.
Huang Foen Chung 2004 [45]	Netherlands	Outpatient clinic	Prospective cohort study	555	MEC is a suitable and reproducible procedure for measuring isovolumetric bladder pressure. Complications associated with MEC include pain in the glans penis (65, 10%), macrohematuria (45, 7%), syncope (3, 0.5%), and hematomas of the skin of the penis (6, 0.9%).
Rajaei-Ishafani 2022 [46]	Iran	Acute care	Randomized controlled trial	150	Bacteriuria was observed in 19 (38%) patients with IUC; in MEC patients, 10 (20%) had bacteriuria in the condom bag design, and 10 (20%) in the sheathed design.
Jaffe 2024 [47]	USA	Acute care	Nurse-driven pre-post study	N/A	The Present on Admission Catheter protocol led to decreases in standardized infection ratio (36%, from 0.845 to 0.543, p=0.01) and IUC standardized utilization ratio (22%, from 0.882 to 0.69, p < 0.05).
Kelly 2023 [48]	USA	Acute care	Nurse-driven pre-post study	N/A	A model was constructed to evaluate infection risk and costs associated with CAUTI based on two prospective studies comparing MEC and IUC. There was a 48% decrease in infection rate among MEC users. The smallest potential per-day cost avoidance was $226 for IUC users and $81 for MEC users.
Kizilbash 2013 [49]	USA	Acute care	Retrospective cohort study	308	MEC use is associated with less bacteremia (OR 0.5, CI 0.22-0.99) but not mortality (OR 1.5, CI 0.84-2.84)
McPhee 2015 [50]	USA		Prospective cohort study	N/A	An aggressive protocol for the removal of IUC from 14 patients with acute SCI yielded 1 UTI for self-catheterization patients and 2 UTIs for MEC patients.
Montgomerie 1987 [51]	USA	Acute care	Prospective cohort study	53	MEC is associated with increased colonization of *Klebsiella pneumonia* in the urethra (41%), penis (41%), perineum (44%), and anus (33%). Colonization is associated with increased length of hospital stay.
Newman 1985 [52]	USA	Renal function laboratory	Prospective cohort study	N/A	MEC users are associated with tissue invasion and GFR worsening. The most common organisms were *E. coli *(n=12; 28%), *P. aeruginosa* (n=9; 19%), *Alpha streptococcus *(n=7; 15%), Klebsiella (n=5; 11%), and *Proteus mirabilis* (n=5; 11%).
Reeths 2020 [53]	USA	Acute care	Nurse-driven pre-post study	N/A	SUR improved from 1.067 in 2017 to 0.964 in 2018 and 0.736 in 2019.
Stelling 1996 [54]	USA	Acute care	Prospective cohort study	80	Between "everyday" and "every other day" MEC users, there is no difference in redness, pressure ulcer (grade 1), excoriation, swelling, UTI, bladder stones, and renal stones
Yoon 2009 [55]	Korea	Acute care	Case report	N/A	45 y/o paraplegic male, using MEC for 10 years, developed a giant fibroepithelial polyp of the glans penis.

Outcomes

The studies reported different outcomes, including CAUTI, AB, CAUTI per 1000 patient days, CAUTI per 1000 device days, IUC utilization ratio or DUR, standardized infection ratio (SIR), standardized utilization ratio (SUR), hospital-acquired pressure injury (HAPI), pain, LOS, skin manifestations, and costs.

Four peer-reviewed abstracts documented improved CAUTI rates per 1,000 device days following a nurse-driven intervention (Table 2) [25-28]. However, due to the unavailability of complete manuscripts and incomplete details on CAUTI rates, a meta-analysis of the CAUTI IRR could not be performed. Similarly, three peer-reviewed abstracts reported an improved IUC utilization ratio; however, a meta-analysis of the IUC utilization ratio was not feasible due to the incomplete data provided in these abstracts [25,26,28].

**Table 2:** Nurse-driven protocols showing the secondary outcomes reported. CAUTI: catheter-associated urinary tract infection; X: decrease in outcome reported; N/A: Not applicable; SUR: standard utilization ratio; SIR: standard infection ratio; HAPI: hospital acquired pressure injury; DUR: device utilization ratio; ICUR: indwelling catheter utilization ratio

Study identifier	Setting	Decrease in the following outcomes after intervention						
		CAUTI/1000 device days	CAUTI/1000 patient days	ICUR	DUR	SUR	SIR	HAPI
Chirca 2018 [25]	Acute care	X	N/A	X	N/A	N/A	N/A	N/A
Fields 2019 [26]	Acute care	X	N/A	X	X	N/A	N/A	N/A
Ghonim 2020 [27]	Acute care	X	N/A	N/A	N/A	N/A	N/A	N/A
Hennessey 2019 [18]	Acute care	N/A	X	N/A	N/A	N/A	N/A	N/A
Jaffe 2024 [47]	Acute care	N/A	N/A	N/A	N/A	X	X	N/A
Kelly 2023 [48]	Acute care	N/A	X	N/A	N/A	N/A	N/A	N/A
Moore 2024 [36]	Acute care	N/A	N/A	N/A	N/A	N/A	X	N/A
Mayes 2020 [28]	Acute care	X	N/A	X	N/A	N/A	N/A	X
Blake 2024 [42]	Acute care	N/A	N/A	N/A	N/A	X	N/A	N/A
Reeths 2020 [53]	Acute care	N/A	N/A	N/A	N/A	X	N/A	N/A

Several manifestations unrelated to UTIs have been reported (Table 3). Two studies indicated that patients using MEC experienced greater comfort than those using IUC [24,29]. Additionally, skin issues, such as penile necrosis, penile skin erosion, and penile ulcers, have been documented in some studies [30-32]. In one abstract, the LOS was reported to be 106 days for MEC and 102 days for IUC [18]. However, it remains unclear how much of these differences in LOS can be attributed solely to the use of MEC, as 32% of the participants in this study were female [18].

**Table 3:** Other secondary outcomes reported. CAUTI: catheter-associated urinary tract infection; HO-UTI: hospital onset urinary tract infection; IUC: indwelling urine catheter; MEC: male external catheter; OR: odds risk; CI: confidence interval; HA: hospital-acquired; LOS: length of stay

Secondary outcome	Findings	Limitations
Bacterial isolates	MEC isolates include *Pseudomonas*, Gram+ bacteria, *Escherichia coli, Klebsiella, Proteus mirabilis, Candida,* and *Aeromonas*, while IUC includes *Pseudomonas*, *Candida*, *E. coli*, and *P. mirabilis *[17,19,23,33,35,37,38,52,54].	Outcomes were based on observational studies.
Urogenital complication	Reported complications in MEC include bladder stones, hydronephrosis, pain in the glans penis, macrohematuria, hematomas, penile strangulation, urinary retention, fibroepithelial polyp, and penile necrosis [22,30,35,44,45,55].	Outcomes were based on observational studies.
Decubitus ulcer	MEC is associated with penile skin ulceration and decubitus ulcers, but a decrease in HA pressure injury [31,35,41,54].	Outcomes were based on observational studies.
Cost-effectiveness	The smallest potential per-day cost avoidance ranged from $226 to $100 for indwelling catheters for CAUTI and non-CAUTI HO-UTI avoidance, respectively, and from $184 to $81 for condom catheters for CAUTI and non-CAUTI HO-UTI reductions, respectively [48].	This outcome was based on an abstract with incomplete details.
Bacteremia	MEC is associated with reduced risk of bacteremia (OR 0.5, CI 0.22-0.99) but not with mortality (OR 1.5, CI 0.84-2.84) [49].	The outcomes were based on chart review.
Pain	Patients using MEC experienced less pain or discomfort than those using IUC [24,29].	There were discrepancies between patient-reported pain and that documented in the medical charts.
Length of stay	LOS for IUC after reinsertion is 102 days, while LOS with MEC is 106 days [18].	This outcome was based on an abstract with incomplete details.

Numerous studies have identified a range of predominant bacterial isolates in male patients with MEC (Table 3). Specifically, *Pseudomonas aeruginosa*, *Klebsiella pneumoniae,* and *Escherichia coli *are more prevalent in patients with MEC. In one study, Gram-positive bacteria were reported to be more common in MEC [17,19,23,33-37]. Conversely, one case report noted *Aeromonas* as the cause of prostatitis [38].

Meta-Analysis

**Figure 2:**
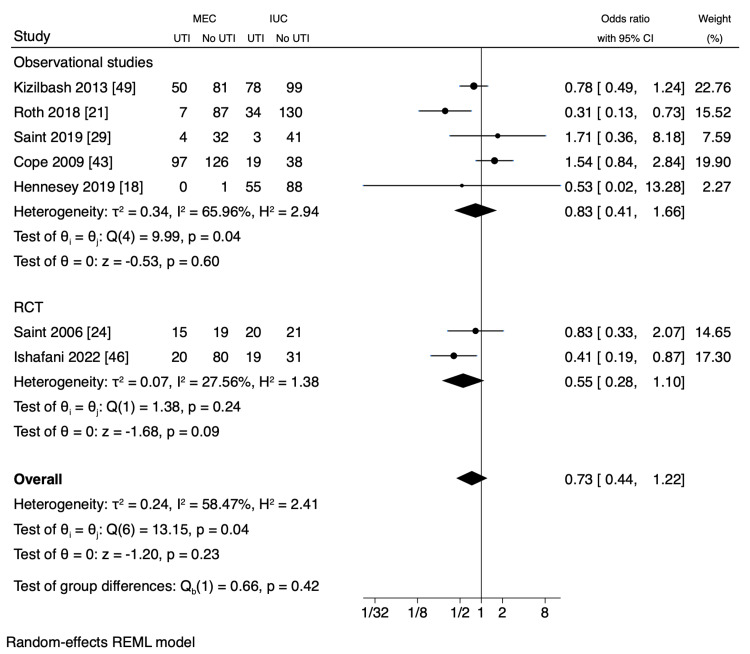
Odds ratio of UTI events between MEC and IUC (less than one favors MEC). MEC: male external catheter; IUC: indwelling urinary catheter; CI: confidence interval; RCT: randomized controlled trial; UTI: urinary tract infection References [18,21,24,29,43,46,49]

**Figure 3:**
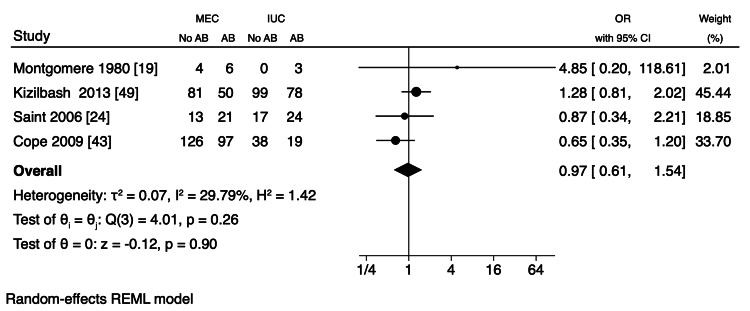
Odds ratio of asymptomatic bacteriuria events between MEC and UTI (less than one OR favors MEC). AB: asymptomatic bacteriuria; MEC: male external catheter; IUC: indwelling urinary catheter; OR: odds ratio; CI: confidence interval; UTI: urinary tract infection References [19,24,43,49]

**Figure 4:**
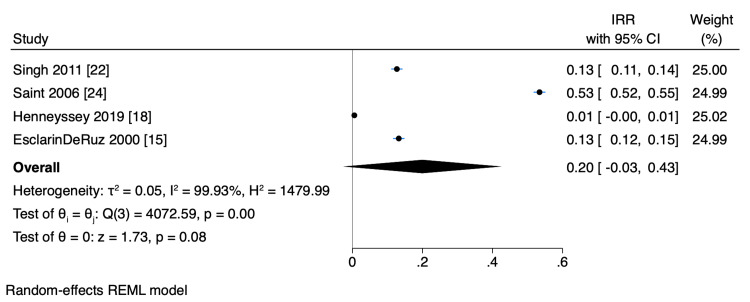
Incidence rate ratio (IRR) between MEC and IUC (less than one favors MEC). MEC: male external catheter; IUC: indwelling urinary catheter; CI: confidence interval References [15,18,22,24]

**Figure 5:**
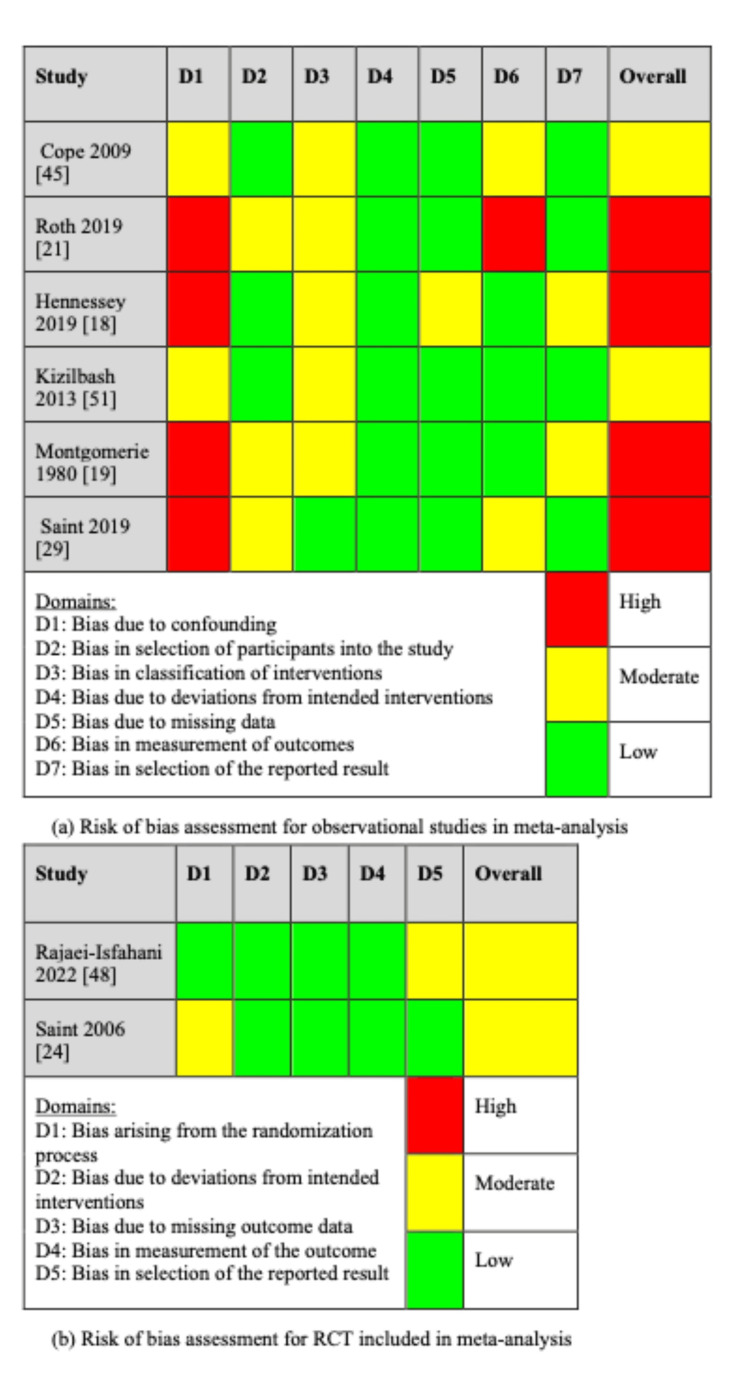
Risk of bias assessment for studies included in the meta-analysis. RCT: randomized controlled trial References [18,19,21,24,29,45,48,51]

A meta-analysis was conducted using data from two RCTs and six observational studies, focusing on MEC-UTI and AB events owing to the comprehensive nature of the methodology, interventional design, and outcomes of these studies (Table 4). Overall, the pooled events of UTI did not show a statistically significant difference between MEC and IUC, although there was a 27% decrease in UTI with MEC (OR 0.73, CI 95% (0.44, 1.22), p=0.23, I^2 ^= 58.47%) (Figure 2). Similarly, there was a 3% decrease in AB with MEC compared to that with IUC, which was also not statistically significant (OR 0.97, CI 95% CI (0.61, 1.54); p=0.90; I^2 ^= 29.79) (Figure 3). Although heterogeneity was moderate in UTI (I^2 ^= 58.47%), less heterogeneity was observed in AB (I^2 ^=29.79%) (Figure 2). Additionally, a sensitivity analysis showed that the pooled UTI events from the two RCTs were not statistically significant between MEC and IUC (OR 0.55, CI 95% (0.28, 1.10), p=0.09; I^2 ^= 27.56%), indicating mild heterogeneity (Figure 2). Furthermore, a meta-analysis revealed a pooled IRR of 0.20 (95% CI, -0.03 to 0.43; p < 0.08, I^2^ = 99.98%) between MEC-UTI and CAUTI (Figure 4). Notably, all studies exhibited a moderate to high risk of bias (Figure 5).

**Table 4:** Characteristics of the studies included in the meta-analysis. X: outcome reported; N/A: not applicable; UTI: urinary tract infection; CAABU: catheter-associated asymptomatic bacteriuria; CAUTI: catheter-associated urinary tract infection; IUC: indwelling urine catheter; MEC: male external catheter; VA: veterans administration

Study identifier	Setting	Study design	Outcomes reported		Limitations
			CAUTI/MEC-UTI	Asymptomatic bacteriuria	
Cope 2009 [43]	Acute care	Retrospective cohort study	X	X	There was an underestimation of patients with asymptomatic bacteriuria treated with antibiotics.
Roth 2019 [21]	Outpatient clinic	Retrospective cohort study	X	N/A	UTI patients were self-reported and not randomized.
Hennessey 2019 [18]	Acute care	Retrospective cohort study	X	N/A	The study was based on an abstract that included a small number of female patients (36, 25%), and only one patient used MEC.
Rajaei-Isfahani 2022 [46]	Acute care	Randomized controlled trial	X	N/A	The results are not generalizable as the participants were Muslim males and circumcised. The study had a small sample size and a short follow-up period.
Kizilbash 2013 [49]	Acute care	Retrospective cohort study	X	X	The classification of CAUTI and CAABU was determined through a review of patient charts.
Montgomerie 1980 [19]	Acute care	Prospective cohort study	N/A	X	Patients were not randomized, and the number of patients using MEC after IUC is unclear. The sample size is small.
Saint 2006 [24]	Acute care, long-term care facility, outpatient clinic	Randomized controlled trial	X	X	There was a small sample size, only one site was used, and it was not possible to blind the patient to the type of catheter.
Saint 2019 [29]	Acute care	Prospective cohort study	X	N/A	The study's findings are not generalizable, as it was conducted in VA hospitals. The sample size is small, and a significant discrepancy exists between patient reporting and medical record reviews.

Discussion

To the best of our knowledge, this study represents the first systematic review and meta-analysis to examine the outcomes of MEC, specifically concerning UTIs and other outcomes (i.e., AB, CAUTI/1000 device days, IUR, dermatologic manifestations, comfort, and bacterial isolates).

This review examines the diverse applications of MEC, focusing on variations in usage contexts, target demographics, and intended purposes. Our findings indicate that these devices are predominantly employed in rehabilitation centers, particularly for younger patients with SCIs. However, their use has also been extended to older males in long-term care facilities. This review underscores the heterogeneity of patient populations utilizing these devices, complicating the identification of a specific group for which they are most suitable. For instance, patients with severe cord compression, spinal cord abnormalities, and urinary retention were excluded from certain studies. This aligns with a consensus study, which suggested that MEC is not recommended for use in specific populations [6].

Several societies, in their latest guidelines (Centers for Disease Control and Prevention; Infectious Diseases Society of America (IDSA, 2018); European Association of Urology, 2024; Agency for Healthcare Research and Quality, 2018), recommend using MEC for specific conditions, such as urinary retention and accurate urinary output monitoring [3,4,56-59]. In patients with psychiatric and neurological conditions, there is no definitive consensus on MEC use, although a higher incidence of UTI has been noted in "uncooperative" patients [24,30,39]. In contrast, patients without dementia who had IUC had a five-fold increased risk of bacteriuria, symptomatic UTI, and death [24]. Finally, risk factors for infection, including age over 60 years, immobility, loss of bladder tone, prostatic hypertrophy, and loss of bactericidal prostatic secretions, may inform the choice of catheterization modality [35,40]. Differences in recommendations underscore the lack of a standardized approach for the use of MEC, highlighting the need for comprehensive guidelines to clarify their appropriate application in specific patient populations.

While this meta-analysis found no statistically significant difference in UTI and AB occurrence between MEC and IUC, these findings must be interpreted cautiously, particularly given the considerable heterogeneity observed for UTI events (I^2 ^= 58.47%). This variability, despite mild heterogeneity in AB (I^2 ^= 29.79%) and the two-RCT sensitivity analysis (I^2 ^= 27.56%), can be attributed to differences in study designs, diverse patient cohorts, and varying clinical environments, limiting the generalizability of pooled results. The relatively small number of studies and limited patient/event numbers for AB further contribute to this caution. Epidemiologically, the "number of events per person-time," such as CAUTI per 1,000 patient-days or 1,000 catheter-days, is the recommended outcome measure rather than the "number of events" alone [3]. In contrast, using the incidence rate, the IRR from our meta-analysis of UTI rates between MEC and IUC was 0.20, suggesting an 80% reduction in UTI incidence in MEC. However, a definitive conclusion cannot be drawn owing to substantial heterogeneity and the limited number of studies with varying effect sizes.

Nurse-driven, protocolized programs aimed at promoting the use of MEC over IUC demonstrated improved CAUTI rates (CAUTI per 1000 device days), as well as enhanced indwelling catheter utilization ratio (IUR). Although these programs were only reported in abstracts, which provided incomplete details about the rates and precluded meta-analysis, the trends in the reported rates indicated a potential benefit of using MEC. These findings suggest that reduced exposure to, or utilization of, IUC correlates with a decreased incidence of CAUTI. Similar findings were observed with FEC; however, evidence supporting this trend in female patients is complete, thus enabling a meta-analysis [9]. Further research is needed to comprehensively investigate the effectiveness of MEC, with a particular emphasis on the impact of nurse-driven protocolized programs.

Although MEC may serve as an alternative to IUC, this review highlights additional secondary outcomes associated with the use of MEC. MEC can lead to bacterial skin colonization of the bladder through the urethra [41]. Dermatological manifestations, including cellulitis, ulcers, penile necrosis, and penile strangulation, have been described (Table 3). Although cases are infrequently reported, this indicates that MEC, despite its seemingly benign nature, is associated with some risks. Overall, the importance of educating healthcare professionals on proper application and monitoring techniques to prevent such complications is essential [29].

Additionally, patient-reported outcomes, such as pain during device removal, have not been consistently documented in medical records, indicating the need for improved documentation practices and patient-centered care [29]. To enhance patient safety and comfort, future studies should implement standardized pain assessment protocols and rigorously adhere to evidence-based guidelines, including the use of validated instruments to monitor dermatological complications and document patient-reported experiences. This approach will facilitate a more comprehensive understanding of the adverse events associated with MEC.

This review highlights the significance of MEC-associated bacterial isolates. Interestingly, the prevalence of *Pseudomonas* has been detected in the catheter, urine bag, perineum, rectum, and surrounding environment of the patient [19,37]. These findings indicate that the widespread presence of these bacterial isolates is likely associated with an increased risk of UTIs in patients with MEC and transmission of infection to other patients [17]. These findings highlight the need for strict infection control and effective catheter management to reduce MEC-UTI and its transmission.

Limitations and Recommendations

Although we successfully outlined the current evidence-based landscape for MEC, we encountered several limitations. First, the variability in study settings, patient characteristics, and inclusion and exclusion criteria across studies posed challenges in generalizing the findings to different populations, significantly affecting comparability. Although this variability mirrors real-world experiences, it also offers an opportunity to standardize outcomes and identify the population best suited for MEC. Additionally, incomplete reporting in some peer-reviewed abstracts limits our ability to conduct comprehensive meta-analyses for certain outcomes. We believe that reporting these abstracts is crucial to providing current evidence that protocolized nurse-driven interventions may help prevent CAUTI.

Furthermore, the increased inconsistency and variability among studies may have contributed to the significant heterogeneity observed in the UTI meta-analysis and the risk of bias in the articles included in the analysis. Notably, most of the included studies were conducted in American healthcare settings, which limits their generalizability to other healthcare settings. The definition of UTI in the included studies may not reflect the current IDSA definition [60]. Finally, we opted to omit a funnel plot, as only eight studies were included in the meta-analysis.

Considering these limitations, we developed important recommendations. First, it is essential to establish a standard definition and criteria for UTIs associated with MEC. This review demonstrated that UTIs do occur, and patients remain at risk, although the level of risk related to MEC is not clearly defined. Given these findings, establishing a standardized approach for UTI documentation is crucial. Adopting this standardized definition will enhance consistency in documenting and reporting UTIs, thereby benefiting future research [9]. Second, we encountered challenges in formulating a generalization and conducting a meta-analysis because of inconsistencies in the reporting of outcomes. Consequently, we recommend adopting standardized outcome reporting to facilitate more comprehensive systematic reviews and meta-analyses. Third, the heterogeneity in methodologies across various studies presents challenges in generalizing the appropriate criteria for the safe use of MEC in specific patient subgroups. Future research should aim to identify the proper indications for use and the specific populations for which MEC is most suitable. Fourth, while the UTI rate is a crucial outcome, our review suggests that other equally significant outcomes are often overlooked. These include considerations such as pain management, length of hospital stays, skin manifestations, bacteriological assessments, and cost-effectiveness of MEC. Fifth, our review revealed a declining trend in the incidence of CAUTI and use of IUC in nurse-driven protocols. We advocate for the development of standardized protocols for MEC application, ensuring they are tailored to specific sites and populations. Finally, future research should focus on conducting large-scale, multicenter RCTs to compare the efficacy and safety of MEC with other catheterization methods across diverse patient populations.

## Conclusions

Although the events of UTIs and AB showed non-statistically significant differences between MEC and IUC, these results should be interpreted with caution because of the considerable heterogeneity observed across studies. This heterogeneity can be attributed to the diverse settings, methodological approaches, and variable populations. While a trend indicated a reduced CAUTI incidence and decreased utilization rates of IUC with nurse-driven protocols, a meta-analysis was not feasible due to incomplete data reporting. To address this limitation, it is recommended that standardized outcome reporting be developed to facilitate more comprehensive systematic reviews and enhance our understanding of the efficacy of MEC.
